# Dynamic time warping analysis of accelerometry data: a tool for interpreting fine-scale movement patterns during fish angling events

**DOI:** 10.1093/conphys/coag034

**Published:** 2026-06-03

**Authors:** Jacey C Van Wert, Stephen D Johnston, Quin V Johnston, Kaitlyn R Zinn, Brian J Hendriks, Lance A Weber, Zachary A Siders, David A Patterson, Kendra A Robinson, Erika J Eliason, Scott G Hinch

**Affiliations:** Institute for Food and Agricultural Sciences, School of Forest, Fisheries, and Geomatic Sciences, Fisheries and Aquatic Sciences Program, University of Florida, PO Box 110410, 1745 McCarty Drive, Gainesville, FL 32611–0410, USA; Pacific Salmon Ecology and Conservation Laboratory, Department of Forest and Conservation Sciences, The University of British Columbia, 2424 Main Mall, Vancouver, BC V6T 1Z4, Canada; Pacific Salmon Ecology and Conservation Laboratory, Department of Forest and Conservation Sciences, The University of British Columbia, 2424 Main Mall, Vancouver, BC V6T 1Z4, Canada; Pacific Salmon Ecology and Conservation Laboratory, Department of Forest and Conservation Sciences, The University of British Columbia, 2424 Main Mall, Vancouver, BC V6T 1Z4, Canada; Pacific Salmon Ecology and Conservation Laboratory, Department of Forest and Conservation Sciences, The University of British Columbia, 2424 Main Mall, Vancouver, BC V6T 1Z4, Canada; Sport Fishing Institute of British Columbia, 19155 38 Ave #104, Surrey, BC V3Z 0Y6, Canada; Institute for Food and Agricultural Sciences, School of Forest, Fisheries, and Geomatic Sciences, Fisheries and Aquatic Sciences Program, University of Florida, PO Box 110410, 1745 McCarty Drive, Gainesville, FL 32611–0410, USA; Fisheries and Oceans Canada, Aquatic Research Cooperative Institute, School of Resource and Environmental Management, Simon Fraser University, 8888 University Drive, Burnaby, BC V5A 1S6, Canada; Fisheries and Oceans Canada, Aquatic Research Cooperative Institute, School of Resource and Environmental Management, Simon Fraser University, 8888 University Drive, Burnaby, BC V5A 1S6, Canada; Fisheries and Oceans Canada, Pacific Science Enterprise Centre, 4160 Marine Drive, West Vancouver, BC V7V 1H2, Canada; Pacific Salmon Ecology and Conservation Laboratory, Department of Forest and Conservation Sciences, The University of British Columbia, 2424 Main Mall, Vancouver, BC V6T 1Z4, Canada

**Keywords:** Bycatch, catch-and-release, fishing, management, Pacific salmon

## Abstract

Post-release survival in catch-and-release fisheries is highly variable and context dependent, yet management agencies often apply uniform survival estimates. A significant source of this uncertainty is our limited understanding of how the capture event itself drives physiological stress and recovery. Fight dynamics reflect how much effort a fish expends and the exhaustion it experiences. Non-invasive accelerometers attached to the fishing line to record the capture event provide a powerful yet underused tool for quantifying these fight dynamics. Traditional analytical approaches reduce accelerometry data to summary statistics, obscuring fine-scale temporal patterns. Here, we introduce a toolbox based on dynamic time warping (DTW) to preserve the temporal structure of capture events and link fight behaviour to physiological disturbance. We attached tri-axial jerk accelerometers (‘jerk’ tags) to fishing lines and captured Chinook salmon (*Oncorhynchus tshawytscha*) and coho salmon (*O. kisutch*) using rod-and-reel angling off the Pacific coast of Vancouver Island in British Columbia, Canada. We quantified physiological disturbance by sampling blood pH and plasma lactate 1 h post-capture. To determine whether biologging tags can predict physiological outcomes, we systematically evaluated 13 analytical pipelines, including jerk summary metrics (fight duration, burst frequency, intensity patterns) and DTW-based approaches using raw, filtered and differentiated jerk data. These complementary approaches captured different behavioural dimensions linking fight to physiology. Summary metrics described broad fight patterns and clustered fish with temporally similar fight signatures, while DTW detected *Y*-axis dimensions linked to individual recovery (plasma lactate). This toolbox is transferable across species and logger types, requiring only accelerometers attached to angling gear. By systematically evaluating processing pipelines rather than defaulting to conventional metrics only, researchers can optimize inference and identify behavioural signatures that predict physiological disturbance. This approach provides a scalable tool for developing evidence-based best practices to improve conservation outcomes.

## Abbreviations

ActVSum: Activity vector sum
CI: Credible interval
CV: Coefficient of variation
DTW: Dynamic time warping
FL: Fork length
GLM: Generalized linear model
ODBA: Overall dynamic body acceleration
PC: Principal component
PCA: Principal component analysis
PCo: Principal coordinate
PCoA: Principal coordinate analysis
RAMP: Reflex action mortality predictors
SD: Standard deviation

## Introduction

Millions of tons of fish are discarded or released annually, representing ~11% of global commercial catch and 60% of recreational catch, respectively (Cooke and Cowx, 2006; Pérez Roda, 2019). Best practices for catch-and-release are designed to maintain fishing opportunities while minimizing population-level impacts (Cowx, 2002; Cooke and Schramm, 2007; Brownscombe *et al.*, 2017). Yet, survival following release is not guaranteed. Post-release mortality estimates span 0%–95%, with considerable inter- and intra-specific variation, even under seemingly similar capture conditions (Bartholomew and Bohnsack, 2005; Prystay *et al.*, 2025). This variability highlights the knowledge gaps in how capture drives survival.

A capture event presents a significant physiological challenge for fish. During angling, fish exert themselves to escape the gear, rapidly exceeding the capacity of aerobic metabolism and forcing a shift to anaerobic pathways, which quickly depletes muscular energy reserves (Cooke and Suski, 2005). This process leads to the accumulation of lactate and protons in their muscles, contributing to metabolic acidosis (Wood, 1991; Wang *et al.*, 1994). In addition, the hook, landing interaction, exposure to air and handling further disrupt their physiology by causing osmoregulatory stress and physical injuries, such as hook wounds, scale loss and fin damage (Ferguson and Tufts, 1992; Nguyen *et al.*, 2014; Cook *et al.*, 2015; Skov *et al.*, 2023; Lunzmann-Cooke *et al.*, 2024; Zinn *et al.*, 2026). Concurrently, the primary stress response triggers the release of stress hormones such as cortisol into the bloodstream (Barton, 2002). These combined disturbances can hinder physiological recovery, compromise post-release swimming performance, increase the fish’s vulnerability to predation or secondary stressors and ultimately lead to mortality (Cooke *et al.*, 2013).

Attention is turning to the measurement of non-lethal onboard indicators of disturbance and condition in fish. Metrics such as reflex action mortality predictors (RAMP), external injury assessments and factors like angling duration, air exposure and handling time have served as proxies to estimate capture severity and predict survival (Arlinghaus *et al.*, 2007; Danylchuk *et al.*, 2007; Davis, 2010; Raby *et al.*, 2012; Cooke *et al.*, 2013; Lennox *et al.*, 2017; Teffer *et al.*, 2017). Experienced anglers recognize that the fight is dynamic and varies across species, individuals, conditions (e.g. current, depth), angling techniques (e.g. casting, jigging, trolling) and gear used (Fedler and Ditton, 1994; Bear and Eden, 2011; Cooke *et al.*, 2021). Recent studies have utilized accelerometers attached to fishing rods (Brownscombe *et al.*, 2014) or fishing lines (Gallagher *et al.*, 2017; Bouyoucos *et al.*, 2018; Knotek *et al.*, 2022) to provide insights into the relationship between fight intensity, duration and recovery. However, reducing high-resolution tri-axial data to summary statistics (fight duration, intensity and maximum tension) disregards the fine-scale temporal structure of the fight and may obscure valuable information (Broell *et al.*, 2013). As research progresses and incorporates physiology, genomics and environmental conditions (Madliger *et al.*, 2018), multidimensional analyses will require advanced tools to visualize, disentangle and leverage this complexity.

Dynamic time warping (DTW) offers a complementary approach that preserves high-resolution data. DTW is a time-series comparison method that quantifies the similarity between sequences by optimally aligning them via ‘warping’, accommodating variation in movement speed and timing (Rakthanmanon *et al.*, 2012). This approach retains fine-scale temporal variation and allows for comparisons among individual fish with different time-series durations. Despite widespread use in data mining (Keogh and Pazzani, 2000) and machine learning (Li and Clifford, 2012), DTW remains underutilized in ecological research (but see Weideman *et al.*, 2017; Hegg and Kennedy, 2021; Siders *et al.*, 2022). Importantly, DTW retains the tri-axial time-series characteristics and preserves nuanced temporal signatures of a fight, often lost in summary statistics.

Here, we introduce a toolbox that provides various pathways for analysing acceleration data (summary and DTW data frames) to quantify movement magnitude and temporal dynamics during capture. Using Pacific salmon (*Oncorhynchus* spp.) as a model system, we pair fine-scale behavioural data with post-capture physiological measurements to test for mechanistic links between capture behaviour and disturbance. We quantify the temporal pattern of jerk acceleration through different analytical methods and assess whether these identify fish with similar fights and predict commonly used physiological indicators of stress (i.e. blood pH and plasma lactate concentrations). By linking behavioural fight signatures to intrinsic traits or environmental and fishery contexts, researchers and managers can use this toolbox technique to inform evidence-based best practices. Although demonstrated here with Pacific salmon in a sport-fishing context, the principles are broadly transferable to other species.

## Materials and Methods

### Site and fishery

The experiment took place on the west coast of Vancouver Island in Barkley Sound within 10 km of Bamfield Marine Sciences Centre (48.835611, −125.135733; Pacific Fisheries Management Area 23, Southern B.C. Salmon Integrated Fisheries Management Plan, IFMP). Salmon fishing is popular in this area and is frequented by recreational charter guides, tourists and First Nations. The primary rod-and-reel recreational angling methods for Pacific salmon in this area are troll downriggers to maintain the gear at desired depths and flashers to attract the fish. These methods mimic those of commercial trollers on a small scale. A downrigger consists of a spool of heavy line attached to a large weight (4.5–8.5 kg) that descends fishing lures to a desired depth (Patterson *et al.*, 2017; Lunzmann-Cooke *et al.*, 2024; Zinn *et al.*, 2026).

### Angling

All handling, care and experimental protocols used were approved by the University of British Columbia (A21-0142) and Bamfield Marine Sciences Centre (RS-22-04-R3). Fish collection was authorized by Fisheries and Oceans Canada (XR 3632024). Adult Chinook [*O. tshawytscha; N* = 11, fork length (FL) = 72.6 ± 7.4 cm; mean ± standard deviation] and coho salmon (*O. kisutch*; *N* = 3, FL = 72.0 ± 3.6 cm) were angled from three recreational vessels with two rods per boat, using downriggers fitted with a 7-kg weight for each rod. Fishing rods were equipped with single action reels (1:1 gear ratio) with 300 m of fishing line (entirely 15-kg monofilament or 24-kg braided nylon with a 15-m segment of 18-kg monofilament to attach to the lure). Lures were limited to either a spoon (7.5–18 cm) or a hoochie skirt (7.5–15 cm), both rigged with a single 2/0 or 3/0 hook (shank-to-tip gap width <15 mm) and attached to an inline flasher (28 cm with reflect tape on both sides). Hoochies were attached using monofilament leader material (15 or 18 kg, 75–100 cm behind the flasher), and spoons were attached using fluorocarbon leader material (15 or 18 kg, 150 cm behind the flasher).

Jerk (*g* s^−1^ or m s^−3^) is the third derivative of position and quantifies motion smoothness. A tri-axial jerk accelerometer tag (MCFT3-3A-A-L; Lotek Wireless Inc., Newmarket, ON, Canada) was attached along the longitudinal *X*-axis to a six-bead chain swivel, secured with wraps of 25-kg braided nylon fishing line and a layer of electrical insulation tape, then placed between the hook (45 cm) and flasher (65–115 cm; Fig. S1). Based on its orientation, the *X*-axis is along the line from the hook to the flasher and represents forward–backward movement, and the *Y*- and *Z*-axes are perpendicular to the *X*-axis. The *Y*-axis represents lateral movement, and the *Z*-axis represents vertical movement; however, since the tag is attached via a swivel, the *Y*- and *Z*-axes may change. The tag sampled jerk acceleration (rate of change of acceleration) at 50 Hz, logging four values every 2 s: mean change in acceleration units along three axes (*X*, *Y* and *Z*) and mean activity vector sum of jerk acceleration (ActVSum) scaled to 0–2*g* s^−1^ at a resolution of 0.4*g* s^−1^. Hardware constraints limited data storage to mean values only, precluding access to internal samples.

Fishing took place from 16 August to 6 September 2024 between 06:30 and 17:00. Angling locations were chosen based on daily fishing reports. Location bathymetry ranged from 18- to 120-m bottom depth, and fish were captured from 7- to 27-m depth. Although depth at hooking can influence fight time, the correlation between line depth and fight time was not significant (*r* = 0.46, *P* = 0.097), and depth was not considered in subsequent analysis. An angling event (fight duration) was timed from the first indication that a fish was hooked (e.g. rod tip movement) until it was netted with a landing net (range: 58–192 s, mean: 123 s). Surface water temperature ranged from 12°C to 18°C.

### Post-angling sampling

Immediately after netting, fish were submerged in an onboard 440-l tank filled with ambient seawater, and the lure was removed while the fish remained in the net (<20 s). Fish were promptly sealed in a respirometer (47.5 l) and sampled for oxygen consumption rate for 1 h. Oxygen consumption results are not used in this study and are therefore not presented here. At 1 h, the fish was removed from the respirometer, and caudal blood was sampled (2 ml, 21 G needle, lithium heparinized BD Vacutainer; BD, Franklin Lake, NJ, USA). This time point was selected to obtain sufficient data on the rapid phase of metabolic recovery while sampling blood when many physiological parameters remain close to peak disturbance (Zhang *et al.*, 2018; Birnie-Gauvin *et al.*, 2023). A fin clip was taken for DNA population identification, and body metrics (FL and girth) were measured (cm). Body mass (kg) was estimated using the length–girth relationship: Mass = (FL × girth^2^)/27 120 (Hinch *et al.*, 2024). Fish were then released at the capture location.

### Blood metrics

Caudal blood samples were immediately measured for pH and then kept in ice slurry and spun within 2 h. Blood pH was measured by placing a pre-calibrated, robust fibre-based pH sensor (Pyroscience, Germany) directly into the Vacutainer. The Vacutainer was then suspended in the ambient tank water alongside a temperature probe (Pyroscience) to ensure the blood pH was measured at the fish temperature. Plasma and red blood cells were separated by centrifugation at 7000*g* for 5 min and frozen at −80°C. Plasma samples were analysed for lactate in duplicate using an L-Lactate analyser (YSI, USA) (Farrell *et al.*, 2001).

### Statistical analyses

**Figure 1:**
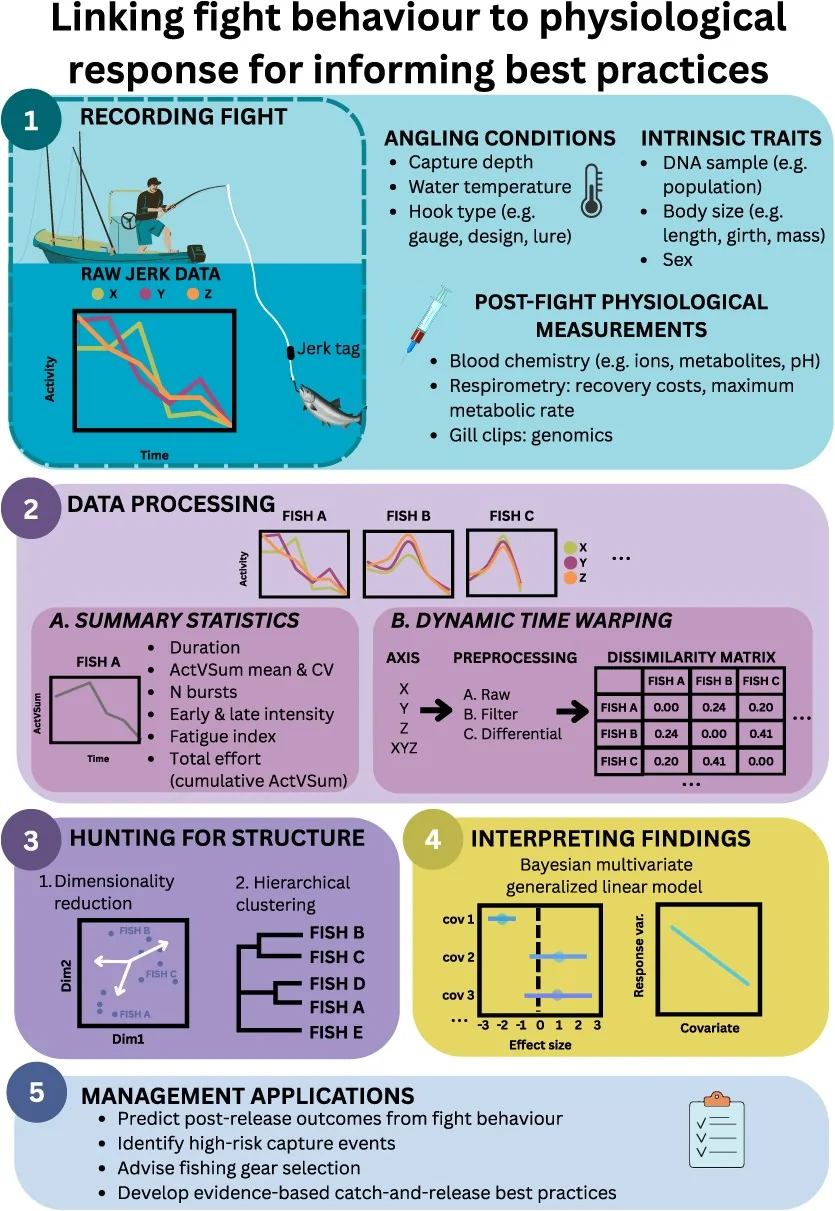
Processing pipeline for jerk accelerometry and physiology data. Traits in box 1 include those not collected here but that can be used in future studies. Statistics calculated in box 2 are presented in Fig. S2. Although applied to jerk data here, the same processing pipeline can be applied to accelerometry data.

Analyses were done in R (version 4.2.1). All values presented are mean ± standard deviation (SD) unless otherwise noted. We evaluated two complementary approaches to characterize fight behaviour from jerk tag data: summary statistics and DTW (Fig. 1). The analysis workflow and visualization tools are available on GitHub (https://github.com/jcvanwert/fightr/).

#### Summary statistics

We assessed the correlation between axes (*X*, *Y* or *Z*) using Pearson’s correlation. Fight behaviour summary metrics were calculated from the integrated jerk statistic reported by the jerk tags, the activity vector sum (ActVSum; *g* s^−1^, where *g* represents standard gravitational acceleration), which is bounded between 0 and 2*g* s^−1^. ActVSum is calculated by the tag as the vectorial magnitude of jerk across all three axes (Text S1) and represents the total intensity of movement regardless of orientation. This metric captures the abruptness of movement changes rather than sustained acceleration. True jerk values (m s^−3^) are represented by multiplying values by the gravitational constant 9.81 m s^−2^ (Lotek MCFT3 Logger Guide).

For each fish, we calculated both basic and novel behavioural metrics (Fig. S2). Basic metrics, including mean ActVSum and its coefficient of variation (CV), total effort (cumulative ActVSum) and number of discrete bursts, were quantified. Burst events were defined as periods when ActVSum exceeded 1.25*g* s^−1^, where the number of discrete bursts was quantified using run-length encoding. This threshold was selected based on preliminary observations and represents a conservative estimate; however, further validation would be needed to establish a definitive value. Fight duration was also calculated, but due to its correlation with other metrics, it was excluded from the principal component analysis (PCA).

To capture behavioural dynamics independent of fight duration, we calculated three novel time-resolved metrics. Early intensity was calculated as the mean ActVSum during the first third of the fight, and late intensity was calculated as the mean ActVSum during the final third. The fatigue index was estimated as the linear rate of change in ActVSum following initial peak effort (requiring ≥3 observations after peak). These metrics provide additional context about how energy expenditure changes throughout the capture event.

#### Dynamic time warping

To compare movement patterns during capture while accounting for variation in fight duration, we employed DTW. This algorithm aligns time series by finding the optimal warping path while minimizing alignment costs (Senin, 2008; Fig. S3). Without a priori expectation for optimal data processing, we systematically evaluated 12 data processing combinations using jerk data from 14 fish: individual jerk axes (*X*, *Y*, *Z*), a multivariate approach incorporating *X*, *Y* and *Z*, a 4-s high-pass filter applied to each axis and its multivariate, the first derivatives of each axis and multivariate derivatives (*dtw* package; Giorgino, 2009). The high-pass filter is intended to isolate the higher frequency components of each signal while the derivative of each jerk axis provides snap (m s^−4^). DTW-based Euclidean distances were calculated between all fish pairs, yielding dissimilarity matrices for each data processing technique.

### Comparing fights across fish

To compare fights across fish, both analytical approaches (summary statistics and DTW dissimilarity matrices) underwent dimensionality reduction: PCA for z-standardized fight metrics and principal coordinate analysis (PCoA) for DTW dissimilarity matrices. The first three dimensions were retained for each (PC1–PC3 for PCA; PCo1–PCo3 for PCoA). For axis-specific DTW analyses (e.g. *X*-axis), PCo1–PCo3 represent orthogonal dimensions of variation in that axis’s temporal patterns, ranked by variance explained. Reduced datasets were then clustered using agglomerative hierarchical clustering with Ward’s *D* algorithm, which is appropriate for Euclidean distance (Murtagh and Legendre, 2014). We determined the optimal number of clusters using the gap statistic with 100 bootstrap replicates (*cluster* package; Maechler *et al.*, 2021). We then selected the smallest cluster number (*k*) for which the gap statistic exceeded the gap statistic for *k* + 1 clusters minus its bootstrap standard error (Siders *et al.*, 2022).

### Linking movement to physiology

To explore relationships among behavioural and physiological variables, Pearson correlations were computed across the number of bursts, fight duration, mean ActVSum, total effort, water temperature, FL, lactate and post-angling blood pH, and results were presented as a correlogram. High collinearity among these variables motivated our use of a multivariate approach. To identify which analytical method best predicted physiological metrics, we fit Bayesian multivariate generalized linear models (GLM) (*brms* package; Bürkner, 2017) with two response variables: post-angling caudal blood pH and plasma lactate. We selected these two metrics because they reflect the severity of anaerobic metabolism and acid–base disturbance during exhaustive exercise, which are expected to scale with the intensity and duration of capture events across fish taxa (Brownscombe *et al.*, 2014; Gallagher *et al.*, 2017; Bouyoucos *et al.*, 2018). The DTW models used the first three principal coordinate (PCo) scores from the respective method as predictors with body mass, water temperature and species as covariates, while the jerk summary model used the first three PC scores with the same covariates plus fight duration. Fight duration was included as a separate covariate for the jerk summary model because these summary statistics collapse temporal information, whereas DTW inherently captures temporal dynamics through sequence-to-sequence comparison. Models assumed Gaussian distributions with identity links. Response variables were normalized (*bestNormalize*; Peterson, 2021) and continuous covariates (body mass, water temperature) were z standardized to enable effect size comparison across methods. Models were fit using four Markov chains, each with 3000 warm-up iterations and 1000 post-warm-up iterations. We assessed model convergence with the Gelman–Rubin statistic 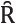 (Gelman and Rubin, 1992) and calculated the 95% credible intervals (CI) of the model intercepts, with credible effects when 95% CI excluded zero. We compared methods using 10-fold cross-validation information criteria (*K*-fold IC; lower = better out-of-sample prediction) and Bayesian *R*^2^ (higher = better in-sample fit). Cumulative variance explained quantifies movement variation captured but does not reflect predictive performance.

For fish *i*:


$$ {y}_i=\left[{\mathrm{physio}}_i\right]={\beta}_o+{\beta}_{\mathrm{dim}}{D}_i+{\beta}_{\mathrm{cov}}{X}_i+{\varepsilon}_i $$

where ${\mathrm{physio}}_i$ = normalized physiology parameter (blood pH, lactate); ${D}_i$ = (Dim1, Dim2, Dim3); ${X}_i$ = (species, water temperature, mass [and fight duration for jerk summary input]) and ${\varepsilon}_i$ is the multivariate residual error term.

Vectors (blood pH and lactate) were fitted onto the ordination using the envfit() function (*vegan* package; Oksanen *et al.*, 2022). Statistical significance was assessed using permutation tests (999 permutations), with vectors considered significant at *P* < .05. Marginal effects of significant predictors were visualized by plotting model predictions across the observed range of each covariate, while holding other variables at their mean values. Predictions were back-transformed to the original scale and plotted with 95% CI.

## Results

**Figure 2:**
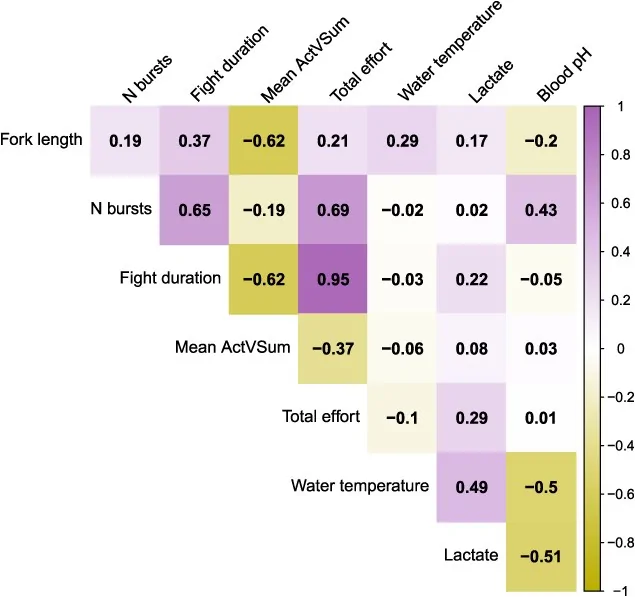
Pearson correlation coefficients between fight metrics, surface water temperature and blood chemistry. Colour intensity represents correlation strength, ranging from negative (yellow) to positive (purple).

A total of 14 fish were captured with jerk tags attached to the line and assessed for blood chemistry at 1 h post-capture. Environmental, behavioural and physiological variables exhibited substantial collinearity (Fig. 2). Among fight metrics, fight duration and total effort were strongly correlated (*r* = 0.95), as well as fight duration and number of bursts (*r* = 0.65), while fight duration had an opposing relationship with mean ActVSum (*r* = −0.62). Larger fish were associated with lower mean ActVSum (*r* = −0.62) but longer fights (*r* = 0.37). Blood pH was negatively associated with plasma lactate (*r* = −0.51), and moderate correlations were observed between fight metrics and physiological variables. Water temperature was positively correlated with lactate (*r* = 0.49) and negatively correlated with blood pH (*r* = −0.50).

### Blood chemistry

Post-angling physiological disturbances varied substantially among individuals (Table S1). Plasma lactate ranged from 10.2 to 22.2 mmol L^−1^ (16.7 ± 3.4 mmol L^−1^) and blood pH ranged from 7.53 to 7.80 (7.67 ± 0.08).

### Comparing fights across fish

**Figure 3:**
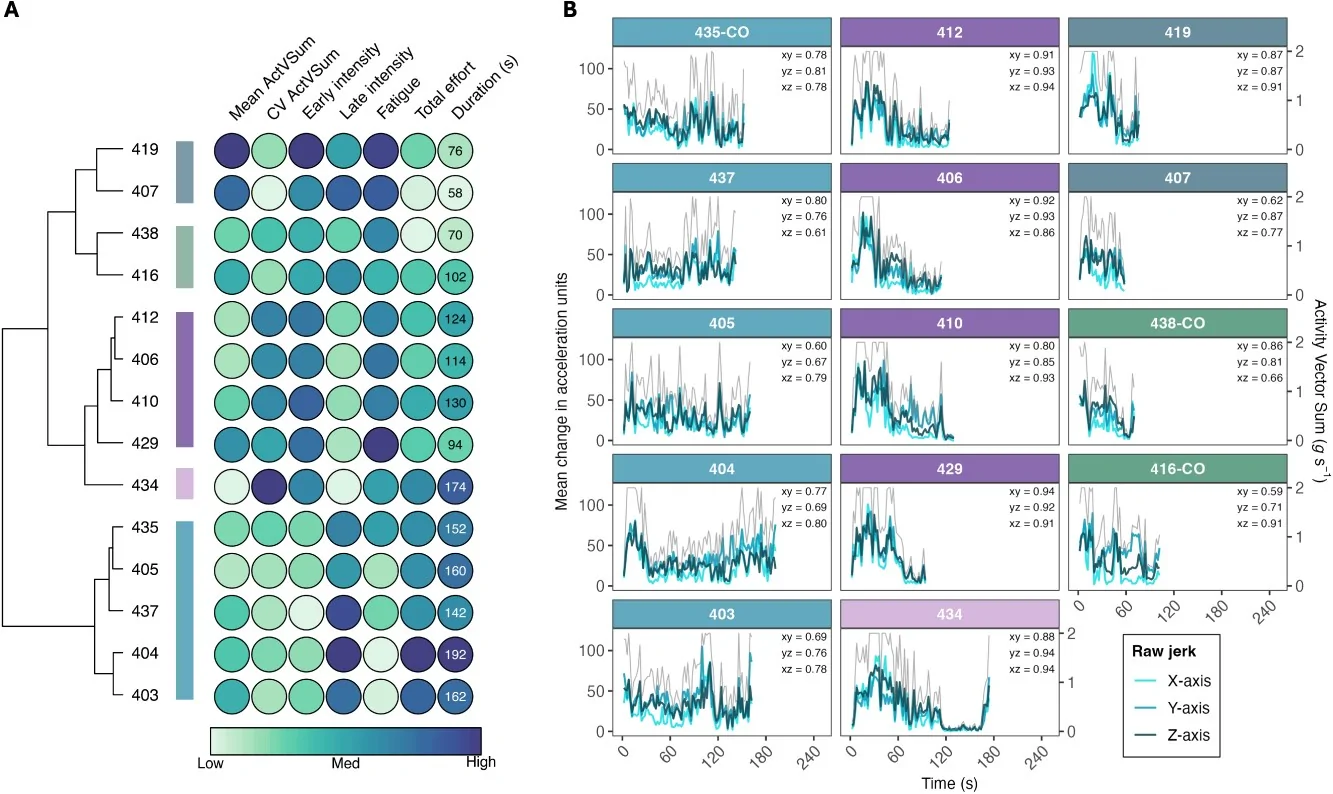
Summary jerk metrics from 14 Pacific salmon. (**A**) The hierarchical clustering between jerk summary metrics for salmon, where each cluster is indicated by colour blocks to the right of the fish id. The metric for each fish is scaled and displayed as a gradient from light green (low) to dark blue (high). Fight duration values (s) are provided. See Fig. S2 for metric calculations. (**B**) The raw jerk data (*X*-, *Y*-, *Z*-axes in turquoises; left *y*-axis) and activity vector sum (ActVSum; grey; right *y*-axis). The time series spans from hook-up (time 0) to netting. Jerk is calculated as the change in acceleration, sampled at 50 Hz and averaged over a 2-s reporting period for each of the three jerk tag axes. The Pearson correlations between *X*-, *Y*- and *Z*-axes are indicated on the top right of each plot. Above each plot is the cluster colour and fish identification, where all fish are Chinook salmon except those indicated with ‘–CO’, which are coho salmon.

Fight duration ranged from 58 to 192 s (Chinook salmon: 130 ± 42 s; coho salmon: 108 ± 41 s). Tri-axial jerk correlations revealed coordinated movement during the angling event (Fig. 3B). Inter-axis correlations (*XY*, *YZ*, *XZ*) ranged from 0.59 to 0.94 across all fish, indicating variable degrees of synchronization in movement across the three axes. Fish that exhibited higher inter-axis correlations (>0.85, fish 412, 406, 410, 429, 419, 434) displayed highly synchronized movements across all three dimensions (mean correlation = 0.90 ± 0.04), suggesting potential directed, coordinated movement. In contrast, fish with moderate correlations (e.g. fish 405, 407, 416, 438) showed lower movement synchronization across axes (mean correlation = 0.72 ± 0.08), indicative of more erratic or complex directional changes.

Jerk summary data also exhibited considerable interindividual variation (Table S2). Chinook salmon performed 3 to 12 discrete bursts, while coho salmon performed 5 to 12 bursts. Mean ActVSum was similar between species, averaging 1.07 ± 0.14*g* s^−1^ for Chinook and 1.04 ± 0.06*g* s^−1^ for coho salmon. The ActVSum CV ranged from 0.37 to 0.82, indicating variable burst consistency among individuals. The ratio of early to late intensity ranged from 0.93 to 1.92, with most fish displaying higher intensity during the earlier part of the fight. Total effort varied 2.8-fold across all individuals, ranging from 35.4 to 100.5*g* s^−1^ and the fatigue index ranged from 0 to 0.5.

The cluster analysis on jerk summary metrics revealed five distinct activity profiles (Fig. 3). Chinook salmon 419 and 407 exhibited the highest activity levels, with intense but brief fights (45 ± 11*g* s^−1^ total effort; 67 ± 13 s duration, respectively). A second cluster showed early intensity and included Chinook salmon 412, 406, 410 and 429 (59 ± 5*g* s^−1^ total effort; 116 ± 16 s duration), while a third cluster with later intensity comprised coho salmon 435 and Chinook salmon 437, 405, 404 and 403 (83 ± 12*g* s^−1^ total effort; 162 ± 19 s duration). Coho salmon 438 and 416 formed a fourth cluster with the lowest overall activity profile (46 ± 15*g* s^−1^ total effort; 86 ± 23 s duration, respectively). Chinook salmon 434 formed its own cluster with primarily early effort and a long duration (77*g* s^−1^ total effort; 174 s duration).

### Linking movement to physiology

We compared 13 movement analysis approaches for predicting post-angling physiological stress (blood pH and plasma lactate) with jerk summary metrics or acceleration data (raw, transformed or filtered; Table S2; Figs S4 and S5). All models showed adequate convergence (maximum 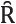 < 1.01) and effective sample sizes (minimum effective sample size ratio >0.18), indicating reliable parameter estimation despite the small sample size (*n* = 14). The wide CIs across the models likely reflect limited statistical power given the small sample size and unbalanced species ratio.

**Figure 4:**
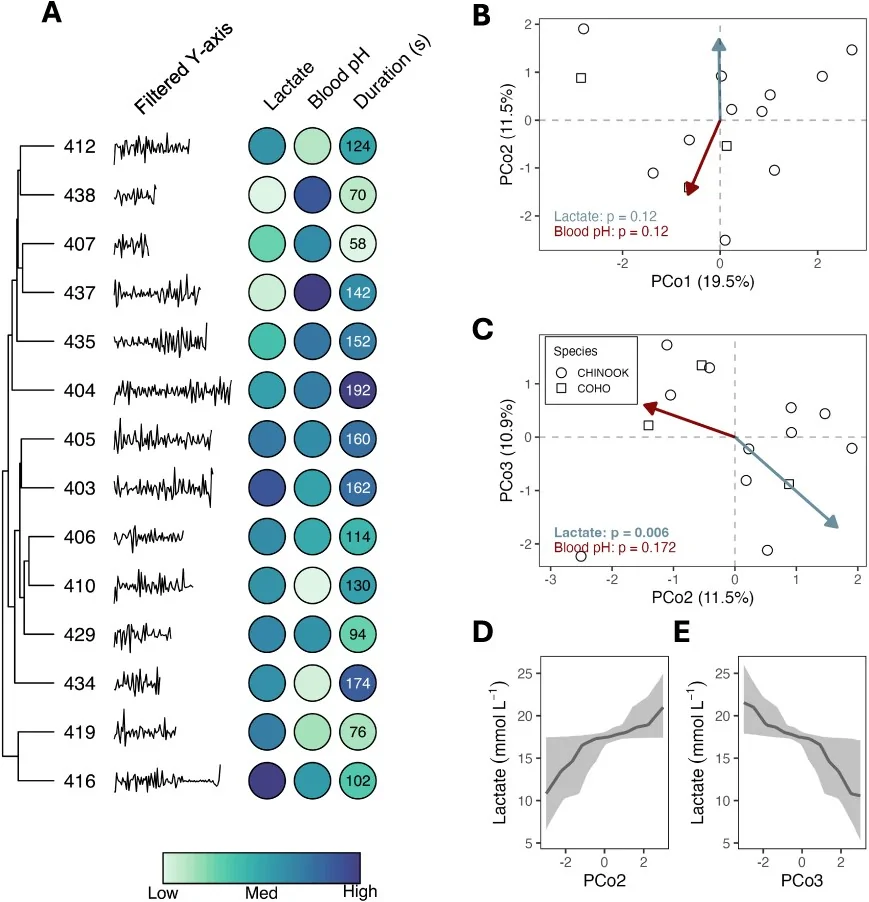
Best performing model using jerk patterns (high-pass filtered *Y*-axis) to predict blood pH and plasma lactate in 14 Pacific salmon. (**A**) The hierarchical clustering of high-pass filtered *Y*-axis jerk patterns for individual salmon. Hierarchical clustering identified no discrete cluster. Physiological metrics for each fish are scaled and displayed as a gradient from light green (low) to dark blue (high). Fight duration values (s) are provided but not included in the hierarchical clustering. Raw traces from the filtered *y*-axis are shown on the left. (**B** and **C**) The first through third PCo from the DTW dissimilarity matrices calculated from the high-pass filtered *y*-axis for each fish. Each point represents an individual fish’s first, second and third PCo score with shape indicating species. Arrows indicate the loadings of each physiological variable on the first, second and third PCo, with *P*-value reported. (**D**) Marginal effects of PCo2 and (**E**) PCo3 on predicted plasma lactate concentrations. Model predictions are demonstrated with 95% CI as shaded regions.

The high-pass filtered *Y*-axis model demonstrated the best model performance, with the lowest *K*-fold information criterion (IC = 95) and one of the highest Bayesian *R*^2^ (0.50). This model explained 41.9% of cumulative variance across the first three PCo dimensions. Unlike jerk summary analysis (Fig. 3A), hierarchical clustering of high-pass filtered *Y*-axis distance matrix identified only a single cluster (Fig. 4A). The high-pass filtered *Y*-axis model was the only one in which jerk axes dimensions significantly predicted physiological outcomes, suggesting the *Y*-axis captured movement patterns relevant to physiology. PCo2 significantly predicted plasma lactate (*β* = 0.41; 95% CI [0.02, 0.79]), with fish exhibiting higher PCo2 scores showing higher post-angling plasma lactate, indicative of severe physiological disturbance (Fig. 4C and D). PCo3 also significantly predicted plasma lactate (*β* = –0.49; 95% CI [−0.85, −0.13]), with fish exhibiting lower PCo3 scores showing higher post-angling plasma lactate (Fig. 4C–E). Neither species, body mass, nor PCo1 significantly predicted blood pH or lactate (Fig. 4B). The residual correlation between blood pH and lactate was weak with a wide CI (*r* = 0.25; 95% CI [−0.38, 0.74]), indicating these physiological responses varied independently.

The jerk summary approach performed the next best (*K*-fold IC = 99.8, *R*^2^ = 0.51) and explained the highest cumulative variance (94.4%), indicating a comprehensive characterization of movement variation. The jerk summary metrics approach yielded no significant predictors. All other jerk tag analysis methods performed substantially worse in terms of predictive accuracy, with *K*-fold values exceeding 104.7 and Bayesian *R*^2^ below 0.38 (Table 1). Notably, the high-pass filtered *X*-axis and 𝑓′(*Y*)-axis yielded a significant predictor for water temperature, where higher water temperature predicted more plasma lactate (*β* = 1.02; 95% CI [0.10, 1.92]) and lower blood pH (*β* = −0.63; 95% CI [−1.26, −0.01]), respectively. No other analytical methods yielded a significant predictor for the covariates.

**Table 1:** Performance comparison of movement analysis methods for predicting post-angling blood pH and plasma lactate.

**Method**	**Cumulative variance (Dim1–Dim3) (%)**	***K*-fold IC**	**Bayesian *R*** ^**2**^	**Sig dim**	**Sig covariates**
High-pass *Y*	41.9	95	0.50	Dim2, Dim3	None
Jerk summary metrics	94.4	99.8	0.51	None	None
High-pass *X*	46.4	104.7	0.37	None	Water temperature
𝑓′(*Y*)	42	107.1	0.38	None	Water temperature
High-pass *XYZ*	35.2	108.3	0.36	None	None
*Z*	52.6	109.6	0.37	None	None
𝑓′(*Z*)	41.2	113	0.36	None	None
𝑓′(*XYZ*)	33.9	115.5	0.31	None	None
High-pass *Z*	41.9	118.3	0.34	None	None
*Y*	51.9	119.7	0.31	None	None
𝑓′(*X*)	43.7	120	0.31	None	None
*X*	61	122.5	0.30	None	None
*XYZ*	47.8	133.4	0.29	None	None

All models included species, sex and body mass as covariates, as well as fight duration for the jerk summary model. Models are ordered by predictive performance [*K*-fold cross-validation IC, lower is better]. Dimensions represent either PC axes from jerk summary metrics or PCo axes from DTW analyses of raw acceleration, high-pass filtered or differential jerk data. Dim = dimension; Sig = significant.

## Discussion

We developed a toolbox for comparing tri-axial-based analytical methods that quantify fight dynamics and link them to physiological outcomes. By evaluating 13 processing pipelines, we demonstrate how analytical choices influence the detection of relationships between fish fights and physiological disturbance. Using Pacific salmon as a model system, we found that the jerk summary metrics identified discrete behavioural clusters, while the dimensions of the filtered *Y*-axis provided the strongest predictions of post-angling plasma lactate levels. Together, these results highlight that various analytical approaches capture distinct components of the fight and can offer insights into the relationships among intrinsic traits, fight patterns and physiological outcomes.

### Summary metrics and DTW as complementary tools in biologging

Our comparison of jerk summary metrics and DTW approaches illustrates two complementary ways to describe fight behaviour. Summary statistics were valuable for characterizing broad behavioural trends. Although jerk summary metrics did not significantly predict blood pH or lactate in our study, they identified behavioural clusters with distinct fight profiles, offering the advantage of being inherently easier to describe, interpret and compare. We calculated both conventional metrics (duration, total effort, number of bursts) and novel metrics designed to capture behavioural dynamics independent of duration (fatigue index, early intensity, late intensity). Many summary metrics correlate with fight duration (Brownscombe *et al.*, 2014; Gallagher *et al.*, 2017; Bouyoucos *et al.*, 2018), which is often associated with low pH and high plasma lactate (Danylchuk *et al.*, 2014; Dapp *et al.*, 2016; Eliason *et al.*, 2022). By including fatigue index and intensities, we expanded the behavioural space beyond duration, enabling the detection of coarse clusters representing distinct fight styles, though our sample size limited statistical power to detect strong correlations between these fight characteristics and physiological variables.

By contrast, DTW preserved temporal architecture by aligning entire jerk sequences and accommodating differences in fight duration. This was particularly effective for jerk-based burst swimming patterns, which contain rapid transitions and irregular structure. Although hierarchical clustering of the high-pass filtered *Y*-axis did not identify discrete behavioural clusters, individuals with similar waveform structure were consistently positioned near one another, indicating meaningful local similarity. DTW methods outperformed summary metrics in predicting physiological state, suggesting that subtle temporal features carry informative signals of exertion and stress.

We selected blood pH and lactate as physiological response variables because (i) they are often associated with traditional fight metrics (fight duration, intensity or maximum acceleration) (Brownscombe *et al.*, 2014; Gallagher *et al.*, 2017; Bouyoucos *et al.*, 2018), (ii) they change predictably in response to exhaustive burst swimming (Milligan and Wood, 1986) and (iii) they allowed us to assess whether fight characteristics independent of duration predict physiological disturbances. Correlations between jerk summary metrics (i.e. number of bursts, fight duration, mean ActVSum and total effort) and these physiological variables revealed weak, inconsistent relationships (Fig. 2). This was confirmed by the multivariate model, which showed no significant relationships for the jerk summary metrics. However, by using multiple components of the jerk signal data via DTW, we found various biological patterns that summary metrics did not detect. This included components of the fight, represented in PCo2 and PCo3, that were associated with lactate levels, as well as high water temperature, which was associated with low blood pH and high plasma lactate levels. Though the mechanisms remain difficult to interpret at this stage and more data are needed for robust conclusions, incorporating more samples and physiological variables may help identify which components of the fight drive physiological outcomes.

### Processing pipelines

Our systematic evaluation of 13 processing pipelines demonstrated that analytical choices influence predictive performance. Jerk (m s^−3^), the third derivative of position, is particularly sensitive to abrupt behavioural transitions such as escape responses. It is not surprising that the *Y*-axis captured a biological relationship since it is perpendicular to the *X*-axis and captured either lateral movement from the fish’s escape response through undulatory swimming (Hawkins  *et al.*, 2025) or downward runs, depending on its position. The *X*-axis, on the other hand, is constrained during a fight and likely reflects the angler’s pull. Surprisingly, the higher-order derivative snap (m s^−4^) of the *Y*-axis also captured behaviourally relevant variation despite the expectation that successive differentiation amplifies noise (Chartrand, 2017). In some cases, axis-specific models outperformed multivariate approaches, suggesting that aggregating axes can obscure informative patterns. An integrated approach combining the *Y*- and *Z*-axes could further capture physiological trends and warrants investigation in future studies.

Tag movement may capture different patterns depending on sampling configuration or attachment method. While the tag internally sampled at 50 Hz, hardware constraints limited data storage to mean values over 2-s logging intervals, which may not resolve fine-scale movements (e.g. tail beats) but is sufficient for detecting overall activity patterns here. Tag rotation during the fight could occur because the tag is attached via a swivel, causing the tag’s orientation relative to the fish to change unpredictably. Rotation would affect individual-axis interpretations and could inflate magnitude estimates if there were rotational forces. Future studies could either examine inter-axial correlations over short time windows to determine if the axes maintain consistent relationships (Noda *et al.*, 2013) or use body-mounted tags or gyroscopes to more precisely quantify or remove rotation effects (Broell *et al.*, 2013, 2016; Noda *et al.*, 2014). Regardless, ActVSum minimizes inter-axial rotational effects by incorporating all three axes, with the caveat that it could overestimate fight intensity if the tag captures rotation beyond the fish’s movement. While fully quantifying these rotation effects is beyond the scope of this study, these considerations underscore our central recommendation that researchers systematically evaluate multiple analytical pathways rather than defaulting to conventional approaches like overall dynamic body acceleration (ODBA) or tri-axial aggregation (Shepard *et al.*, 2008).

### Applications

We provide a transparent, reproducible workflow for transforming raw tri-axial data into physiological predictions, applicable beyond our specific study system. The workflow is designed to be modular, allowing researchers to substitute different dimensionality reduction techniques, clustering algorithms or predictive models depending on their research questions and data structures. This flexibility is critical for modern physiological studies integrating multiple data streams (e.g. genomics, proteomics, blood physiology) (Madliger *et al.*, 2018), where collinearity among variables is common. For such datasets, response variables can be reduced to orthogonal PCs capturing integrated responses, which then serve as response variables in regression models to uncover movement–physiology relationships.

A key strength of this toolkit is its performance under realistic field conditions. We used standard recreational angling techniques to catch fish in the wild at varying depths and times of day, thereby introducing heterogeneity in baseline physiological state and activity levels. Field biologging inherently involves trade-offs between experimental control and real-world applicability (Turko *et al.*, 2023), and our results demonstrate that DTW workflows can identify biologically relevant patterns despite uncontrolled conditions. Similarly, the fights recorded here (58–192 s) were relatively short compared to those of other species, which can last over an hour (Gallagher *et al.*, 2017). While our results yielded wide CIs reflecting both limited power and biological variability, they demonstrate that this comparative framework can identify meaningful patterns even with time-limited data.

Linking fight characteristics to physiological outcomes offers a pathway towards evidence-based best practices. If specific fight signatures consistently predict severe physiological compromise, these could inform on-site release decisions, advise fishing gear selection, identify high-risk scenarios and guide management practices that improve conservation outcomes (Cooke and Suski, 2005). Future studies with larger, taxonomically diverse samples may reveal species-, size-, sex- or population-specific response patterns (Eliason *et al.*, 2011, 2020; Van Wert *et al.*, 2023). This approach has utility for research measuring fish exertion and physiological responses, including swim performance tests at high flows, field fish passage evaluations, predator avoidance trials and other studies of anaerobic exercise or stress responses (Brownscombe *et al.*, 2014; Burnett *et al.*, 2014; Wright *et al.*, 2014). The framework may even provide a means of evaluating behavioural types in novel situations (Lennox *et al.*, 2023), offering insights into individual variation in stress-coping strategies. As biologging technologies become increasingly accessible (Hawkes *et al.*, 2021), deploying this approach across broader scales would facilitate the characterization of risk profiles (e.g. size, sex or certain fight characteristics and behaviours) and inform conservation strategies across fisheries.

## Conclusions

DTW provides a powerful approach for analysing tri-axial time series by retaining fine-scale temporal structure and uncovering fight signatures linked to physiological disturbance. By combining summary statistics with DTW analysis, our toolbox identifies behavioural signatures that predict severe physiological compromise. Because this approach requires only attaching accelerometers to fishing gear, it is transferable across fisheries and species, offering a flexible, scalable tool to support evidence-based catch-and-release guidelines and conservation strategies.

## Supplementary Material

Web_Material_coag034

## Data Availability

The data and code underlying this article are available on GitHub (https://github.com/jcvanwert/fightr/).
