# Correction to: Dynamic time warping analysis of accelerometry data: a tool for interpreting fine-scale movement patterns during fish angling events

**DOI:** 10.1093/conphys/coag067

**Published:** 2026-08-25

This is a correction to: Jacey C Van Wert, Stephen D Johnston, Quin V Johnston, Kaitlyn R Zinn, Brian J Hendriks, Lance A Weber, Zachary A Siders, David A Patterson, Kendra A Robinson, Erika J Eliason, Scott G Hinch, Dynamic time warping analysis of accelerometry data: a tool for interpreting fine-scale movement patterns during fish angling events, *Conversation Physiology*, Volume 14, Issue 1, 2026, coag034, https://doi.org/10.1093/conphys/coag034

In the originally published version of this manuscript, flight time duration was given as ‘57-177 s’ when it should have been ‘58-192 s’. This happened in three instances in the article, on pages 3, 6, and 10.

This error has been corrected.

