# Supplementary material for: Dynamic time warping analysis of accelerometry data: a tool for interpreting fine-scale movement patterns during fish angling events
Source: Conserv Physiol. 2026 Jun 3;14(1):coag034. doi: 10.1093/conphys/coag034 (PMC13231511; doi:10.1093/conphys/coag034)
Supplement: Web_Material_coag034 [file web_material_coag034.zip › ConsPhysToolbox_VanWert_supp_revisions.docx]

**Dynamic time warping analysis of accelerometry data: a tool for interpreting fine-scale movement patterns during fish angling events**

Jacey C. Van Wert, Stephen D. Johnston, Quin V. Johnston, Kaitlyn R. Zinn, Brian J. Hendriks, Lance A. Weber, Zachary A. Siders, David A. Patterson, Kendra A. Robinson, Erika J. Eliason, Scott G. Hinch

*Conservation Physiology*

**Text S1**. The activity vector sum (ActVSum) is calculated as the vectorial sum of three axes:

𝐴𝑐𝑡 − 𝑉𝑆𝑢𝑚 [𝑔/𝑠] = √{(𝐴𝑐𝑡 − 𝑋)2 + (𝐴𝑐𝑡 − 𝑌)2 + (𝐴𝑐𝑡 − 𝑍)2}

The values are provided to users as processed (ActVSum). See Lotek MCFT3 Logger Guide for more information.

**
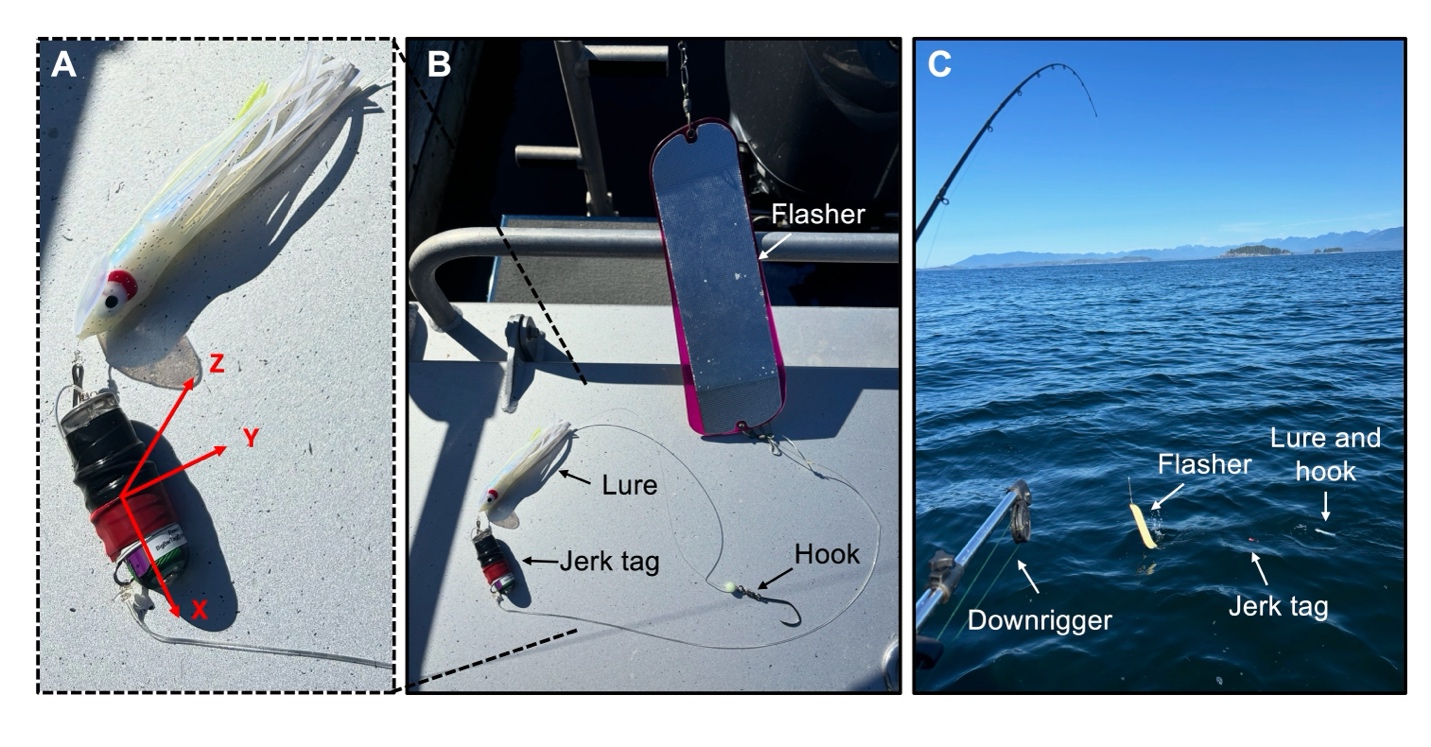
**

**Figure S1**. Rod-and-reel angling setup with jerk accelerometer instrumentation. (A) Orientation of tri-axial jerk accelerometer tag (MCTF3-3A-A-L) attached along the longitudinal X-axis to a 6-bead chain swivel. (B) Complete rigging configuration showing an in-line flasher, jerk tag, adjustable hoochie lure, and hook. (C) Deployment of instrumented fishing gear in the ocean.

**
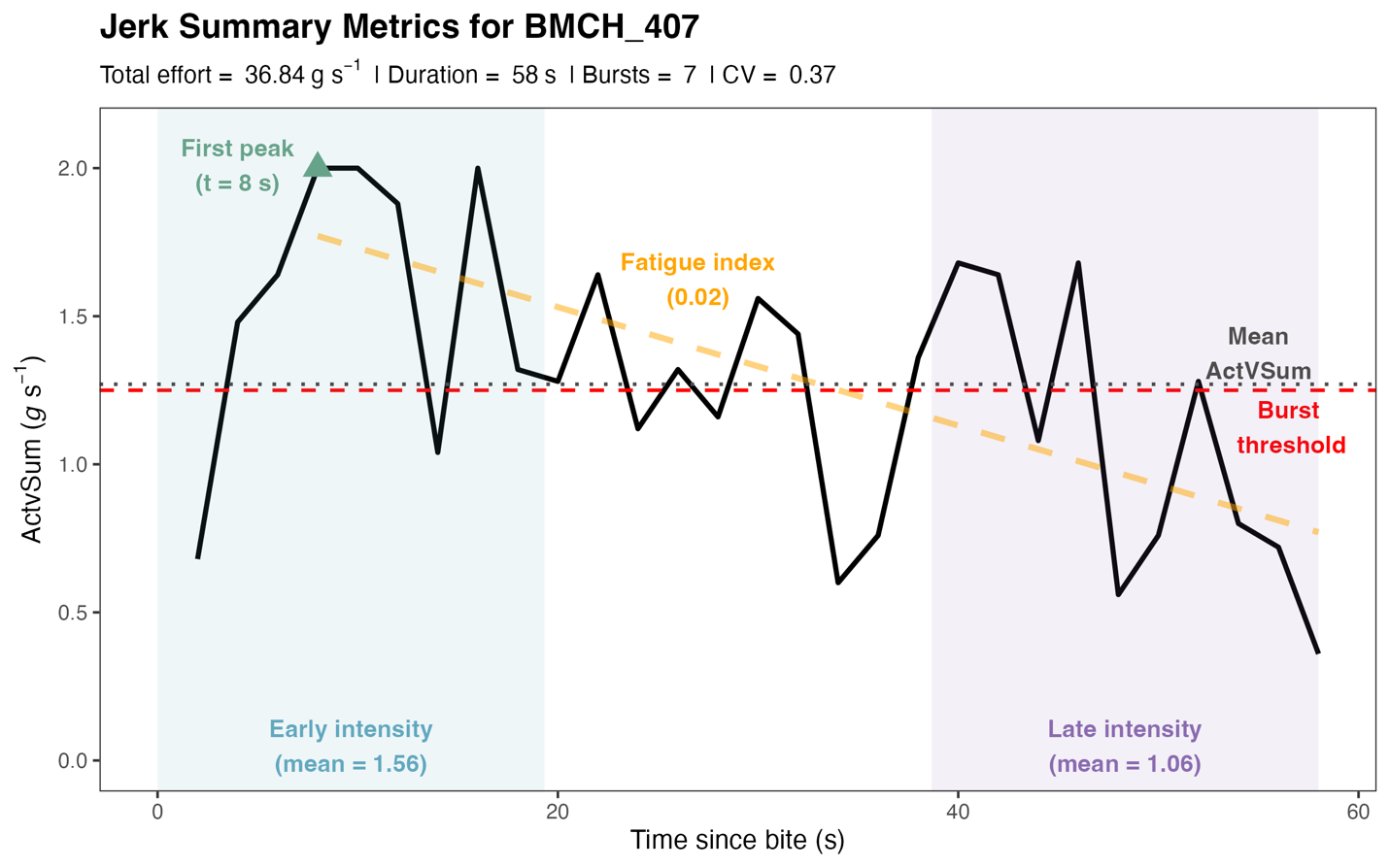
Figure S2**. Example trace from Chinook salmon 407 showing jerk summary metrics. The time series displays ActVSum throughout the fight. Burst events were defined as periods exceeding 1.25 *g* s^-1^. Early intensity was calculated as the mean activity during the first third of the fight, and late intensity was calculated as the mean activity during the final third. The fatigue index was estimated as the linear rate of change in activity following peak effort (requiring ≥3 observations after peak). Total effort was calculated as the cumulative ActVSum.

**
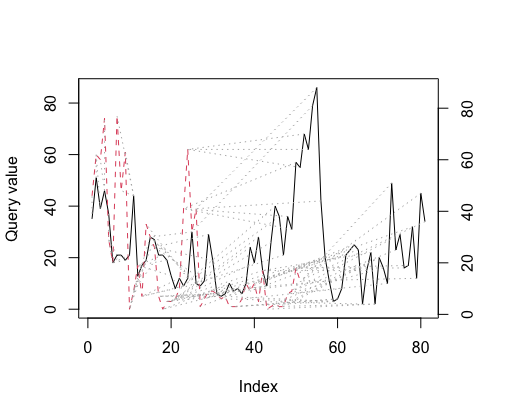
**

**Figure S3.** Alignment of two time-series produced from the dtw package (Giorgino, 2009). The left axis (403-Chinook; black solid) and right axis (416-Coho red dashed) for raw jerk tag X-axis data.


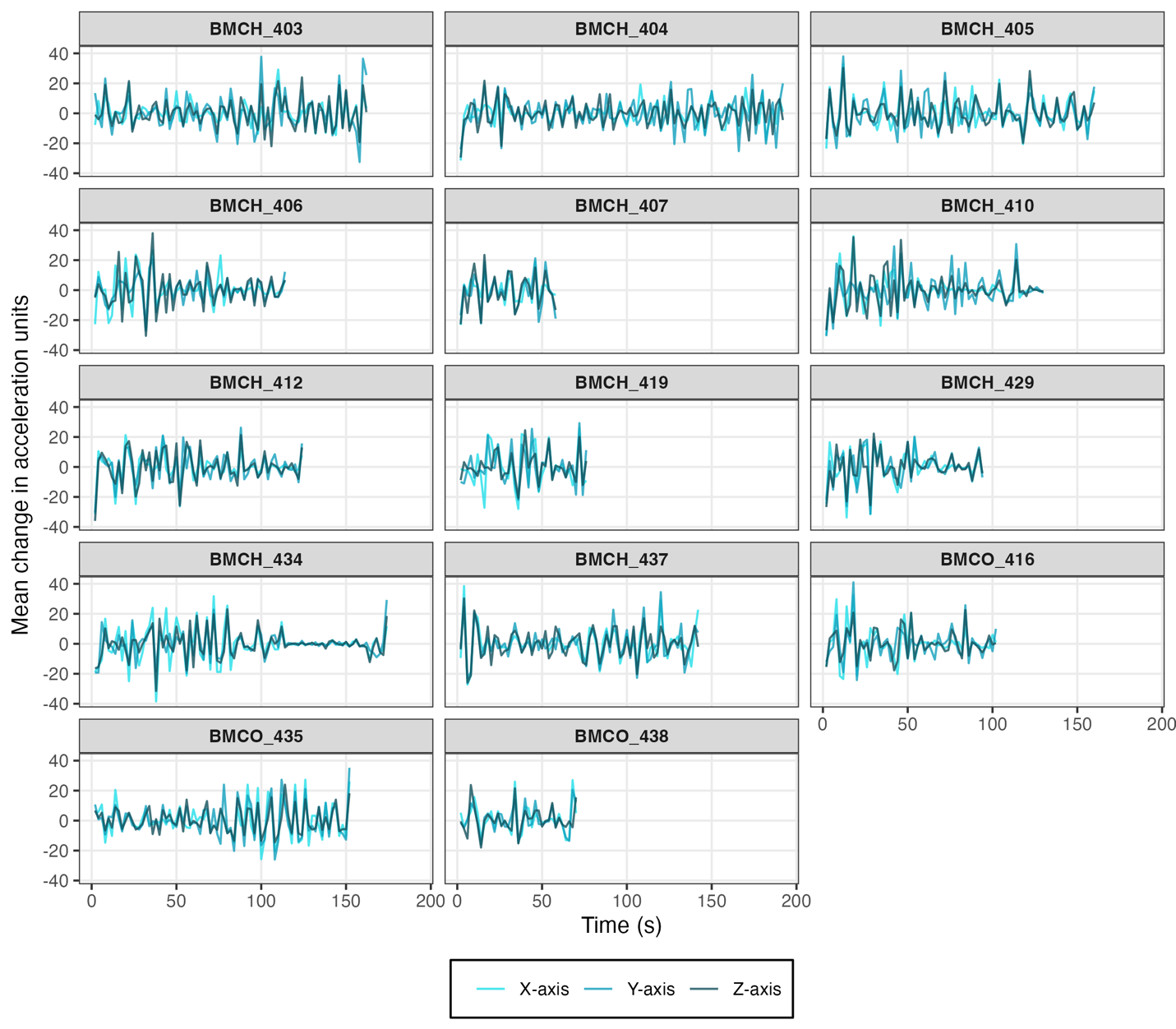


**Figure S4**. The X, Y, and Z-axis jerk data of the fourteen Pacific salmon are shown with 4 s high-pass filtering applied. The time series spans from hook-up (time 0) to netting.


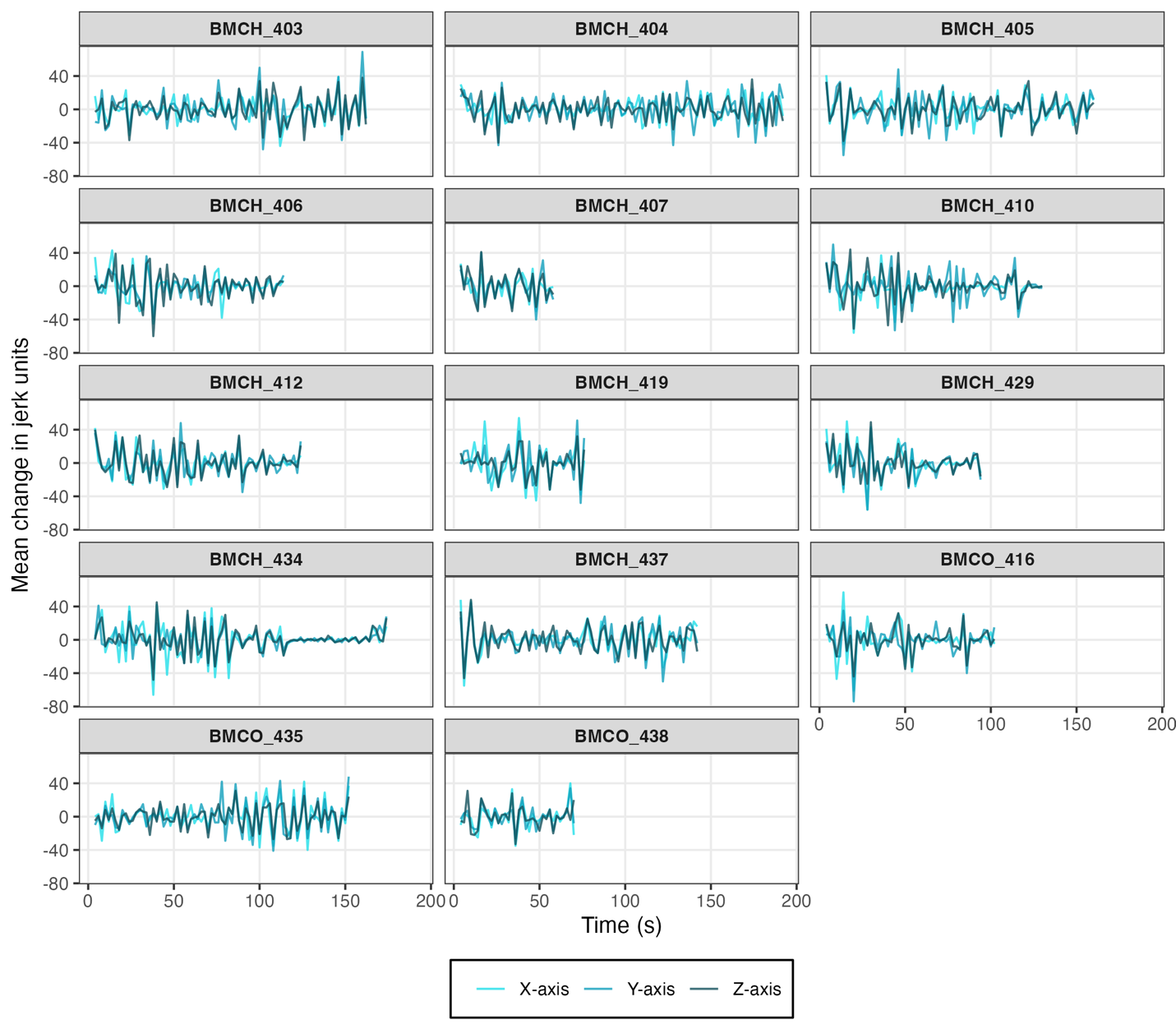


**Figure S5.** The differential of X, Y, and Z-axis jerk data (mean change in jerk units) of the fourteen Pacific salmon. The time series spans from hook-up (time 0) to netting.

**Table S1:** Surface water temperature, traits, and 1 h post-capture blood physiology measured in the fourteen Pacific salmon.

| **Species** | **ID** | **Surface water temperature (°C)** | **Sex** | **FL (cm)** | **Lactate (mmol L^-1^)** | **Blood pH** |
| --- | --- | --- | --- | --- | --- | --- |
| Chinook | 403 | 18.3 | F | 75 | 21.0 | 7.67 |
|  | 404 | 13.1 | M | 64 | 16.6 | 7.72 |
|  | 405 | 17.9 | M | 81 | 18.9 | 7.70 |
|  | 406 | 17.9 | M | 71 | 17.8 | 7.66 |
|  | 407 | 18.1 | M | 64 | 13.4 | 7.70 |
|  | 410 | 16.9 | F | 70 | 17.3 | 7.53 |
|  | 412 | 16.8 | F | 88 | 17.3 | 7.56 |
|  | 419 | 16.0 | M | 65 | 18.6 | 7.57 |
|  | 429 | 15.2 | F | 71 | 18.0 | 7.69 |
|  | 434 | 17.1 | M | 76 | 17.5 | 7.54 |
|  | 437 | 12.4 | M | 74 | 10.8 | 7.80 |
| Coho | 416 | 15.5 | F | 71 | 22.2 | 7.68 |
|  | 435 | 16.8 | M | 76 | 14.7 | 7.73 |
|  | 438 | 13.8 | M | 69 | 10.2 | 7.77 |

**Table S2:** Summary jerk statistics assessed from the (ActVSum) for the fourteen Pacific salmon. Burst events were defined as periods exceeding 1.25 *g* s^-1^. Early intensity was calculated as the mean activity during the first third of the fight, and late intensity was calculated as the mean activity during the final third. Fatigue was estimated as the linear rate of change in activity following peak effort (requiring ≥3 observations after peak). Total effort was calculated as the cumulative ActVSum.

| **Species** | **Fish ID** | **Duration (s)** | **N bursts** | **Mean ActVSum (*g* s^-1^)** | **CV ActVSum** | **Early intensity** | **Late intensity** | **Fatigue Index** | **Total effort**  **(*g* s^-1^)** |
| --- | --- | --- | --- | --- | --- | --- | --- | --- | --- |
| Chinook | 403 | 162 | 11 | 1.10 | 0.43 | 1.18 | 1.00 | 0 | 89.04 |
|  | 404 | 192 | 11 | 1.05 | 0.47 | 1.12 | 1.23 | 0 | 100.48 |
|  | 405 | 160 | 12 | 0.94 | 0.44 | 1.14 | 0.80 | 0.01 | 75.36 |
|  | 406 | 114 | 3 | 0.95 | 0.65 | 1.61 | 0.41 | 0.03 | 54.12 |
|  | 407 | 58 | 7 | 1.27 | 0.37 | 1.56 | 1.06 | 0.04 | 36.84 |
|  | 410 | 130 | 3 | 1.02 | 0.66 | 1.76 | 0.44 | 0.03 | 66.16 |
|  | 412 | 124 | 5 | 0.96 | 0.67 | 1.65 | 0.47 | 0.03 | 59.36 |
|  | 419 | 76 | 5 | 1.37 | 0.45 | 1.92 | 0.76 | 0.05 | 52.08 |
|  | 429 | 94 | 4 | 1.18 | 0.59 | 1.69 | 0.39 | 0.05 | 55.52 |
|  | 434 | 174 | 8 | 0.89 | 0.82 | 1.59 | 0.25 | 0.02 | 77.00 |
|  | 437 | 142 | 9 | 1.04 | 0.43 | 0.93 | 1.16 | 0.01 | 74.04 |
| Coho | 416 | 102 | 7 | 1.11 | 0.45 | 1.41 | 0.85 | 0.02 | 56.72 |
|  | 435 | 152 | 12 | 1.00 | 0.49 | 1.17 | 0.91 | 0.02 | 75.68 |
|  | 438 | 70 | 5 | 1.01 | 0.53 | 1.37 | 0.52 | 0.03 | 35.40 |
